# Amniotic membrane transplantation for infectious keratitis: a systematic review and meta-analysis

**DOI:** 10.1038/s41598-021-92366-x

**Published:** 2021-06-21

**Authors:** Darren Shu Jeng Ting, Christin Henein, Dalia G. Said, Harminder S. Dua

**Affiliations:** 1 Academic Ophthalmology, Division of Clinical Neuroscience, School of Medicine, University of Nottingham, Nottingham, NG7 2RD UK; 2 Department of Ophthalmology, Queen’s Medical Centre, Nottingham, UK; 3 National Institute for Health Research (NIHR) Biomedical Research Centre At Moorfields Eye Hospital NHS Foundation Trust and UCL Institute of Ophthalmology, London, UK

**Keywords:** Microbiology, Medical research, Therapeutics, Diseases, Eye diseases, Infectious diseases

## Abstract

Infectious keratitis (IK) is the 5th leading cause of blindness globally. Broad-spectrum topical antimicrobial treatment is the current mainstay of treatment for IK, though adjuvant treatment or surgeries are often required in refractory cases of IK. This systematic review aimed to examine the effectiveness and safety of adjuvant amniotic membrane transplantation (AMT) for treating IK. Electronic databases, including MEDLINE, EMBASE and Cochrane Central, were searched for relevant articles. All clinical studies, including randomized controlled trials (RCTs), non-randomized controlled studies and case series (n > 5), were included. Primary outcome measure was time to complete corneal healing and secondary outcome measures included corrected-distance-visual-acuity (CDVA), uncorrected-distance-visual-acuity (UDVA), corneal vascularization and adverse events. A total of twenty-eight studies (including four RCTs) with 861 eyes were included. When compared to standard antimicrobial treatment alone, adjuvant AMT resulted in shorter mean time to complete corneal healing (− 4.08 days; 95% CI − 6.27 to − 1.88; *p* < 0.001) and better UDVA (− 0.26 logMAR; − 0.50 to − 0.02; *p* = 0.04) at 1 month follow-up in moderate-to-severe bacterial and fungal keratitis, with no significant difference in the risk of adverse events (risk ratio 0.80; 0.46–1.38; *p* = 0.42). One RCT demonstrated that adjuvant AMT resulted in better CDVA and less corneal vascularization at 6 months follow-up (both *p* < 0.001). None of the RCTs examined the use of adjuvant AMT in herpetic or Acanthamoeba keratitis, though the benefit was supported by a number of case series. In conclusion, AMT serves as a useful adjuvant therapy in improving corneal healing and visual outcome in bacterial and fungal keratitis (low-quality evidence). Further adequately powered, high-quality RCTs are required to ascertain its therapeutic potential, particularly for herpetic and Acanthamoeba keratitis. Future standardization of the core outcome set in IK-related trials would be invaluable.

## Introduction

Corneal infection or infectious keratitis (IK) is the most common cause of corneal blindness worldwide, particularly in the developing countries^1^. The incidence was estimated at 2.5–799 per 100,000 population/year^1^. It is a painful and potentially blinding ocular emergency that often requires hospital admission for intensive medical and/or surgical treatment. Depending on the geographical and temporal variations and population-based risk factors (e.g. agricultural practice, trauma, use of contact lens and others), bacteria and fungi have been shown to be the main causative microorganisms for IK, followed by viruses, parasites and polymicrobial infection^2–7^. Broad-spectrum topical antimicrobial therapy is currently the gold standard for managing IK in routine clinical practice, though eye-saving procedures such as corneal gluing, therapeutic photoactivated chromophore-corneal cross-linking (PACK-CXL), amniotic membrane transplantation (AMT), and therapeutic keratoplasty may be required in recalcitrant cases^8–14^.

Amniotic membrane (AM) is the innermost layer of the placenta, which consists of a single layer of metabolically active epithelium, a thick basement membrane, and an avascular stromal matrix^15^. It has been shown to exhibit a wide array of biological properties, including wound healing, anti-inflammatory, antimicrobial, and anti-angiogenic properties, amongst others^8^. In addition, the wide availability of AM donor tissues, lack of graft rejection and improvement in the storage methods have rendered AMT a popular choice of treatment for ocular surface diseases. So far, AMT has been utilized to treat neurotrophic keratopathy, IK, corneal perforation, limbal stem cell deficiency, chemical eye injury, radiation keratopathy, bullous keratopathy, and many other ocular surface conditions^8,16–23^.

To date, a number of studies have evaluated the benefit of AMT for treating active IK, though the majority of them were of small case series or case reports. In clinical practice, AMT is usually reserved as a second-line therapy in IK, mainly to promote cornea healing in non-healing ulcer after the sterilization phase. Therefore, the value of employing AMT in addition to standard antimicrobial treatment (SAT) during the active phase of IK remains uncertain. Liu et al^24^ recently conducted a systematic review of 17 studies on the use of AMT for infective and non-infective corneal ulcers. Although the review provided a detailed analysis on the corneal healing rate and visual improvement rate associated with the use of AMT, it did not compare the effect of adjuvant AMT and SAT with SAT alone. Furthermore, the study did not include several important relevant studies, including three randomized controlled trials (RCTs)^25–27^ and one non-randomized controlled study (NRCS)^28^, rendering the robustness of evidence uncertain. In light of these limitations, we conducted a systematic review and meta-analysis to critically examine the effectiveness and safety of combined AMT and SAT versus SAT alone in treating patients with IK.

## Results

### Literature search and study characteristics

**Figure 1:**
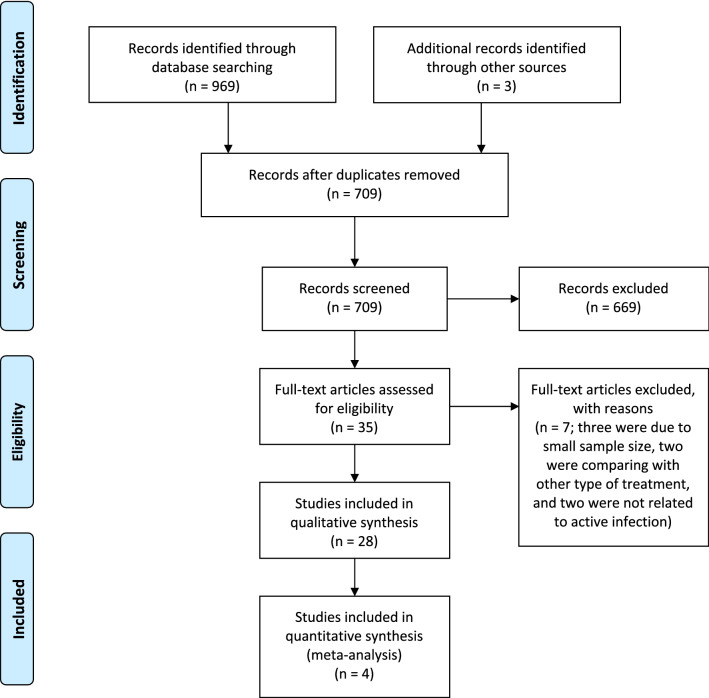
PRISMA flow diagram of the literature search for assessing the evidence of amniotic membrane transplantation for infectious keratitis.

The electronic searches last conducted on 01 November 2020 retrieved a total of 969 titles and abstracts (see Fig. 1 for the PRISMA flow chart). After removing 262 duplicates and including three additional records identified through other sources, the remaining 709 records were screened and 669 references that were not relevant to the scope of the review were excluded. A total of 35 full-text copies of papers were assessed for eligibility. After excluding seven ineligible articles, 28 studies were included in the systematic review. These included four RCTs^25–27,29^, three NRCSs^28,30,31^, and 21 case series^32–52^, examining the efficacy and safety of AMT in 861 eyes with IK, of which 666 eyes received combined AMT with SAT and 195 eyes received SAT alone.

The causative microorganisms in the treatment group of AMT with SAT included 240 (36.0%) fungi, 199 (29.9%) bacteria, 152 (22.8%) herpes viruses, 9 (1.4%) Acanthamoeba, 18 (2.7%) mixed, and 48 (7.2%) unspecified IK (either bacteria, fungi or mixed infection). The main characteristics of all RCTs and NCRSs, including the authors’ name, year of publication, number of treated eyes, types of causative microorganisms, severity of IK, types and techniques of AMT, main outcomes, and adverse events are summarized in Tables 1 and 2, respectively. Outcomes of RCTs are analyzed and summarized under the meta-analysis section.

**Table 1:** Summary of all randomized control trials evaluating the effectiveness and safety of adjuvant amniotic membrane transplantation (AMT) for infectious keratitis.

Authors	Year	Protocol registration*	Age, years	Male gender	Total eyes (AMT)	Total eyes (control)	Causative organisms (in AMT group)				
							B	F	A	V	M
Arya et al.^21^	2008	N	Mean = 41.8 (AMT) versus 50.8 (control)	33 (83%)	20	20	5	12	0	0	3
Li et al.^33^	2014	N	Mean = 43.8 (AMT) versus 47.0 (control)	36 (72%)	25	25	0	25	0	0	0
Tabatabaei et al.^20^	2017	N	Mean = 48.3 (AMT) vs. 43.4 (control)	46 (46%)	49	50	49	0	0	0	0
Zeng et al.^22^	2014	N	Mean = 57.1 (AMT) vs. 54.0 (control)	14 (70%)	10	10	0	10	0	0	0

Authors	Baseline vision (LogMAR)		Time from first presentation to AMT	AMT technique		Severity of ulcer^$^		Follow-up (months)		COI	
Arya et al.^21^	< 20/400 = 80% (AMT) versus 65% (control)		NR	Overlay (single layer)		2–5 mm = 60% (AMT) versus 45% (control)		3		N	
Li et al.^33^	< 20/200 = 44% (AMT) versus 48% (control)		2 days	Overlay (single or double layer)		NS		1		N	
Tabatabaei et al.^20^	Mean = 1.7 (AMT) versus 1.8 (control)		2–5 days	Inlay (double layer)		Mean = 26 mm^2^ (AMT) versus 27 mm^2^ (control)		6		N	
Zeng et al.^22^	Median = 1.0 (AMT) versus 1.0 (control)		NR	Overlay (single layer)		Mean = 7 mm (AMT) versus 7 mm (control)		1		N	

*B* bacteria; *F* fungi, *A* acanthamoeba, *V* viruses, *M* mixed infection, *COI* conflict of interest, *NR* Not reported.

*Prospective registration of the clinical trial protocol in a publicly accessible database.

^$^Severity of the corneal ulcer is presented either in maximum linear diameter (mm) or in area (mm^2^).

**Table 2:** Summary of all clinical studies (excluding RCTs) evaluating the use of amniotic membrane transplantion (AMT) for infectious keratitis.

Authors	Year	Study design	Total eyes (AMT)	Total eyes (control)	Causative organisms (in AMT group)						Severity of ulcer^$^
					BK	FK	VK	AK	MK	NSK	
Kheirkhah et al.^23^	2012	NRCS	14	11	14						Mean = 32% (AMT) versus 33% (control)
Li et al.^34^	2014	NRCS	53	45		53					Not reported
Naeem et al.^35^	2004	NRCS	34	34						34	> 3 mm
Altay et al.^36^	2016	Case series	84	–	42		42				3 mm
Berguiga et al.^37^	2013	Case series	5	–	1		4				Central, deep ulcer/perforation (< 2 mm)
Bourcier et al.^38^	2004	Case series	6	–				6			Stromal lesions
Chen et al.^39^	2006	Case series	23	–		12			11		2–13 mm; > 50% depth to perforation
Chen et al.^40^	2002	Case series	6	–	6						Mean = 29 mm^2^, depth of 25–33% (50%), descemetocele (50%)
Eleiwa et al.^41^	2020	Case series	5	–		5					Perforated corneal ulcer (3–5 mm)
Eraslan Yusufoglu et al.^42^	2013	Case series	46	–	21		25				Paracentral and central ulcer; depth of > 50% in viral (56%) and bacterial (33%) cases
Fu et al.^43^	2012	Case series	35	–		35					Median = 3–5 mm
Gicquel et al.^44^	2007	Case series	12	–	12						Mean = 5 mm
Hoffmann et al.^45^	2013	Case series	12	–	3		5		4		4–20mm^2^, depth of 10–90%
Kim et al.^46^	2001	Case series	21	–	9	2	7	3			Not reported
Li et al.^47^	2010	Case series	18	–			18				< 5 mm (67%), > 5 mm (33%), perforation (6%)
Mohan et al.^48^	2014	Case series	28	–	28						Not reported
Rao et al.^49^	2012	Case series	21	–		21					Not reported
Shi et al.^50^	2007	Case series	15	–			15				20–45mm^2^, depth of 20–33%
Spelsberg et al.^51^	2008	Case series	12	–			12				–
Wan & Huo^52^	2010	Case series	35	–	9	20	6				Not reported
Wu et al.^53^	2013	Case series	18	–			18				1–7 mm
Xie et al.^54^	2014	Case series	19	–		19					3–6 mm
Yildiz et al.^55^	2008	Case series	14	–						14	Not reported
Zhang et al.^56^	2010	Case series	26	–		26					Median = 3–6 mm; 5 perforation

Authors	Preop vision*	Time to AMT	AMT technique**	Combined with SAT	Outcomes (i.e. healing rate and time)		Adverse event***	Postop vision*		Follow-up (months)	
Kheirkhah et al.^23^	2.0 (AMT) versus 2.0 (control)	2–3 days	Overlay (Single)	Y	Complete healing = 100%, Mean healing time = 13 days (AMT) versus 16 (control)		None	Mean = 0.5 (AMT) versus 0.7 (control)		11	
Li et al.^34^	0.6	Not reported	Inlay	Y	Mean healing time = 23 days (AMT) and 35 days (control)		Not reported	Mean = 0.3 (AMT) versus 0.4 (control)		2	
Naeem et al.^35^	Not reported	Not reported	Not reported	Y	Complete healing = 96% (AMT) versus 30 (87%) (control)		Not reported	Not reported		2	
Altay et al.^36^	Majority ≤ 1.0	2–5 days	Overlay (Single or double)	Y	Complete healing = 100% (BK) and 95% (VK), Mean healing time = 19 days		5% (VK)	Improved in vision = > 50% (BK); 2% (VK)		15	
Berguiga et al.^37^	Mean = 1.0	7 days	Mixed (Multiple)	Y	Complete healing = 100% (BK) and 75% (VK)		25% (VK)	Improved in 40% cases		15	
Bourcier et al.^38^	All ≤ CF	Not reported	Inlay or overlay (Single–multiple)	Y	Complete healing = 67%		None	Improved in 50%		14	
Chen et al.^39^	CF–LP	Not reported	Inlay (Single or double)	Y	Complete healing = 87%, Mean healing time = 16 days		13%	20/20-LP		21	
Chen et al.^40^	HM–LP	21 days	Inlay (Single)	Y	Complete healing = 83%, Mean healing time = 9 days		17%	Improved in 83%		13	
Eleiwa et al.^41^	Median = CF	12 days	Inlay (Double)	Y	Complete healing = 100%, Mean healing time = 26 days		None	Median = 0.3		14	
Eraslan Yusufoglu et al.^42^	Median = ≤ 20/200	Not clear	Overlay (Double)	Y	Complete healing = 100% (BK), 88% (VK), Mean healing time = 23 days		12% (VK)	Improved in 57% (BK) and 8% (VK)		12	
Fu et al.^43^	Median = < 20/200	Not reported	Overlay (Double)	Y	Complete healing = 94%, Mean healing time = 14 days		3%	Improved in 94%		12	
Gicquel et al.^44^	Median = ≤ 6/60	2 days	Overlay or mixed (Single–multiple)	Y	Complete healing = 100%, Mean healing time = 26 days		None	Median = 20/40		8	
Hoffmann et al.^45^	Median = 20/20,000	Not reported	Mixed (Multiple)	Y	Complete healing = 83%, Mean healing time = 24 days		17%	Median = 20/200		22	
Kim et al.^46^	20/40—LP	Not reported	Inlay or overlay (Single–multiple)	Y	Complete healing = 100%		None	Improved in 76%		18	
Li et al.^47^	~ 20/120	~ 1 week	Overlay (Double)	Y	Complete healing = 100%		None	Improved in 78%		3–18	
Mohan et al.^48^	< 20/200	4 weeks	Mixed (Multiple)	Y	Complete healing = 75%		25%	Improved in 7%		6	
Rao et al.^49^	Not mentioned	~ 2 weeks	Mixed (Multilayer)	Y	Complete healing = 76%		10%	Not reported		3	
Shi et al.^50^	Median = 20/400	2 weeks	Overlay (Multiple)	Y	Complete healing = 100%, Mean healing time = 15 days		None	Median = 20/40 Improved in 93%		9	
Spelsberg et al.^51^	Median = < 6/60	Not reported	Inlay (Single)	Y	Complete healing = 75%, Mean healing time = 25 days		25%	Improved in 8%		7	
Wan and Huo^52^	Not reported	Not reported	Mixed (Multiple)	Y	Complete healing = 94%		6%	> 20/200 = 83%		1–2	
Wu et al.^53^	> 20/200 = 7 (39%)	1–3 days	Mixed (Multiple)	Y	Complete healing = 100%, Mean healing time = 17 days		None	> 20/200 = 72%		4	
Xie et al.^54^	Mean = 1.3	1 week	Mixed (Double)	Y	Complete healing = 42%, Mean healing time = 36 days		Not reported	Mean = 0.9		3	
Yildiz et al.^55^	Not reported	Not reported	Overlay (Single)	Y	Complete healing = 100%		Not reported	Not reported		22	
Zhang et al.^56^	Not reported	Not reported	Mixed (Double)	Y	Complete healing = 81%		8%	Improved in 80%		3–36	

*BK* bacterial keratitis, *FK* fungal keratitis, *VK* viral keratitis, *AK* acanthamoeba keratitis, *MK* mixed keratitis, *NSK* non-specified keratitis, *SAT* standard antimicrobial treatment, *NRCS* non-randomized controlled studies, *CF* counting fingers, *HM* hand movement, *PL* perception of light.

^$^Severity of the corneal ulcer is presented either in maximum linear diameter (mm), in area (mm^2^) or in percentage of total cornea (%).

*Vision is presented in either Snellen vision or logMAR vision.

**AMT technique is categorized by the type of grafting (inlay as graft vs. overlay as patch vs. mixed inlay/overlay) and number of layers (single layer vs. double layer vs. multiple layers).

***Adverse event was defined as uncontrolled/worsening infectious keratitis requiring tectonic keratoplasty or evisceration.

### Meta-analysis of eligible RCTs

#### Overall description

Four eligible RCTs were included in the meta-analysis, which included a total of 209 eyes that compared the effectiveness of SAT and adjuvant AMT with SAT alone^25–27,29^. These consisted of four single-centre RCTs conducted separately in China (n = 2)^27,29^, India (n = 1)^26^, and Iran (n = 1)^25^. Two additional RCTs were also identified but were excluded from the meta-analysis as one RCT compared AMT with conjunctival flap^53^ and another RCT compared AMT and argon laser treatment with AMT for treating IK^54^. There was no ongoing trial identified from the clinical trial registry databases. The RCTs included participants with an average age between 45.5 and 55.6 years (ranged 15–98 years) with a slight (61.7%) male preponderance. None of the RCTs were prospectively registered with any clinical trial database.

#### Types of microbes and severity of IK

The four RCTs (n = 209 eyes) included a total of 108 (51.7%) eyes with bacterial keratitis, 95 (45.5%) eyes with fungal keratitis and 6 (2.9%) eyes with mixed bacterial/fungal keratitis. No herpetic or Acanthamoeba keratitis was included in any of the RCTs. Li et al.^29^ and Zeng et al.^27^ included only fungal keratitis and Tabatabaei et al.^25^ included only bacterial keratitis whereas Arya et al.^26^ included a mixed cohort, which included bacterial, fungal or mixed bacterial/fungal keratitis. The proportion of the types of microorganisms was similar between the treatment and control groups among all RCTs.

The baseline reporting and extent of the severity of IK were heterogeneous among the four RCTs. Two studies reported the baseline ulcer diameter or area in continuous values^25,27^, one study reported the baseline severity in categorical values (i.e. 1–2 mm, 2–5 mm, or > 5 mm in diameter)^26^, and one study did not report the baseline severity of IK^29^. Tabatabaei et al.^25^ and Zeng et al.^27^ included IK cases with a mean ulcer diameter of 5.2 mm and 6.7 mm, respectively (i.e. severe IK based on previous studies)^55,56^. Similarly, Arya et al.^26^ included mainly moderate-to-severe IK, with 47.5% of moderate ulcer (2–5 mm in diameter) and 42.5% of severe ulcer (> 5 mm in diameter). None of the RCTs included eyes presented with threatened or actual corneal perforation.

#### Surgical technique and timing of AMT

In terms of the preservation technique, two RCTs utilized cryopreserved AMs^26,27^, one RCT utilized freeze-dried AMs^29^, and one did not report the type of AMs used. Three RCTs^26,27,29^ employed AMT as an overlay patch (single- or double-layer) whereas one RCT^25^ employed AMT as an inlay graft (double-layer). All AMTs were performed with the epithelium/basement membrane side up. The time interval between the initial presentation of IK and AMT was 3 days in one study^29^, 2–5 days in one study^25^, and unspecified in two studies^26,27^.

#### Risk of bias

**Figure 2:**
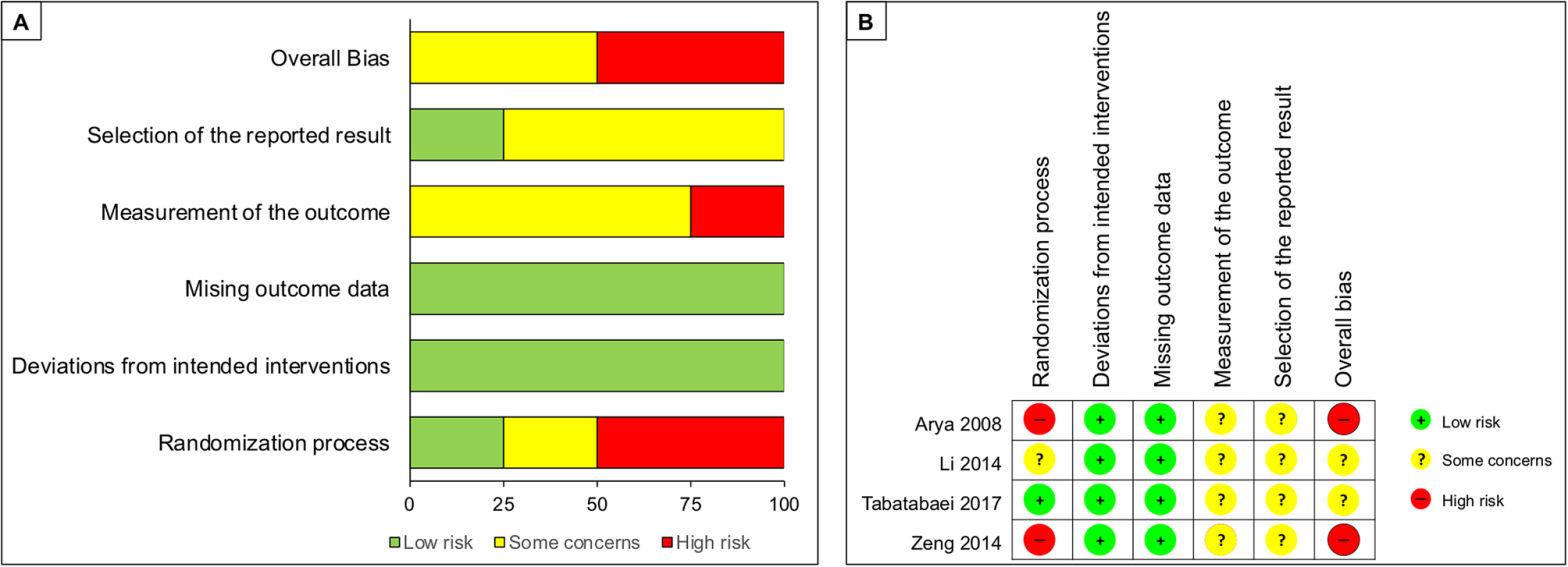
Risk of bias assessment of all included randomized controlled trials (RCTs) based on revised risk of bias tool (RoB 2). (**A**) A summary of review authors' judgements about each risk of bias item presented as percentages across all included RCTs. (**B**) Review authors’ judgements about each risk of bias item presented individually for all included RCTs.

The risk of bias of the RCTs, based on the five abovementioned domains, is summarized in Fig. 2. Only one RCT^25^ clearly reported the randomization process, with details on randomization and allocation concealment. All RCTs had complete or nearly complete (> 99%) follow-up data. There were some concerns about the measurement of the outcome as it was not possible to mask the outcome assessor (i.e. AMT would be visible on postoperative clinical examination, hence revealing the intervention arm). In addition, there were some concerns about selective reporting of outcomes across all RCTs as none of them were prospectively registered with any publicly accessible clinical trial database; therefore, the risk of bias could not be confidently evaluated. No relevant conflict of interest was declared in any of the RCTs.

The risk of bias of NRCS was assessed using the ROBIN-I tool. Two NRCS^28,31^ were judged to be at serious risk of bias due to bias in selection of participants and measurement of outcomes. One NRCS^30^ was judged to be at critical risk of bias due to baseline confounding, bias in selection of participants and measurement of outcomes.

#### Effects of intervention

The effects of interventions were categorized into: (A) time to complete corneal healing (defined by complete corneal re-epithelialization and resolution of infection); (B) UDVA and CDVA (in logMAR) at 1–6 months follow-up; (C) size of ulcer or infiltrate; (D) extent of corneal neovascularization at 1–6 months; and (E) adverse events defined by worsening IK, endophthalmitis or corneal perforation requiring corneal gluing, tectonic keratoplasty or evisceration during the follow-up period. The GRADE summary of findings for each treatment outcome is summarized in Table 3.

**Table 3:** GRADE summary of findings for various treatment outcomes of adjuvant amniotic membrane transplantation for infectious keratitis.

Adjuvant amniotic membrane transplantation (AMT) compared to standard antimicrobial treatment (SAT) for infectious keratitis					
**Patient or population**: Infectious keratitis **Intervention**: Adjuvant amniotic membrane transplantation (AMT) **Comparison**: Standard antimicrobial treatment (SAT)					
Outcomes	Anticipated absolute effects* (95% CI)		Relative effect (95% CI)	No of eyes (studies)	Certainty of the evidence (GRADE)
	Risk with SAT	Risk with AMT			
Time to complete healing (days)	10.2–30.7 days	MD 4.08 days shorter (6.27 shorter to 1.88 shorter)	–	169 (3 RCTs)	⨁◯◯◯ VERY LOW^a,b,c^
UDVA (logMAR) at 1 months	1.27–1.88 logMAR	MD 0.26 logMAR better (0.50 better to 0.02 better)	–	119 (2 RCTs)	⨁◯◯◯ VERY LOW^a,b,c^
CDVA (logMAR) at 6 months	1.55 logMAR	MD 0.43 logMAR better (0.68 better to 0.17 better)	–	99 (1 RCT)	⨁⨁◯◯ LOW^b,c^
Size of corneal scar (mm^2^) at 6 months	22.8 mm^2^	MD 5.17 mm^2^ smaller (7.53 smaller to 2.8 smaller)	–	99 (1 RCT)	⨁⨁◯◯ LOW^b,c^
Corneal vascularization (%) at 6 months	7%	MD 4% smaller (6 smaller to 3 smaller)	–	99 (1 RCT)	⨁⨁◯◯ LOW^b,c^
Adverse events at 1–6 months	23 per 100	18 per 100 (11–32)	RR 0.80 (0.46–1.38)	209 (4 RCTs)	⨁◯◯◯ VERY LOW^a,b,c,d,e^

GRADE Working Group grades of evidence.

High certainty: We are very confident that the true effect lies close to that of the estimate of the effect.

Moderate certainty: We are moderately confident in the effect estimate: The true effect is likely to be close to the estimate of the effect, but there is a possibility that it is substantially different.

Low certainty: Our confidence in the effect estimate is limited: The true effect may be substantially different from the estimate of the effect.

Very low certainty: We have very little confidence in the effect estimate: The true effect is likely to be substantially different from the estimate of effect.

Explanations.

^a^High risk of bias due to lack of randomization and allocation concealment in ≥ 50% of the included studies.

^b^Potential risk of bias due to the lack of blinding in participants and assessors.

^c^The total number of participants is less than the number generated by a conventional sample size calculation.

^d^There are few events and the confidence interval includes appreciable benefit and harm.

^e^Differences in final follow-up duration.

*CI* confidence interval, *MD* mean difference, *RR* risk ratio, *UDVA* uncorrected-distance-visual-acuity, *CDVA* corrected-distance-visual-acuity.

*The risk in the intervention group (and its 95% confidence interval) is based on the assumed risk in the comparison group and the relative effect of the intervention (and its 95% CI).

##### Time to complete corneal healing

**Figure 3:**
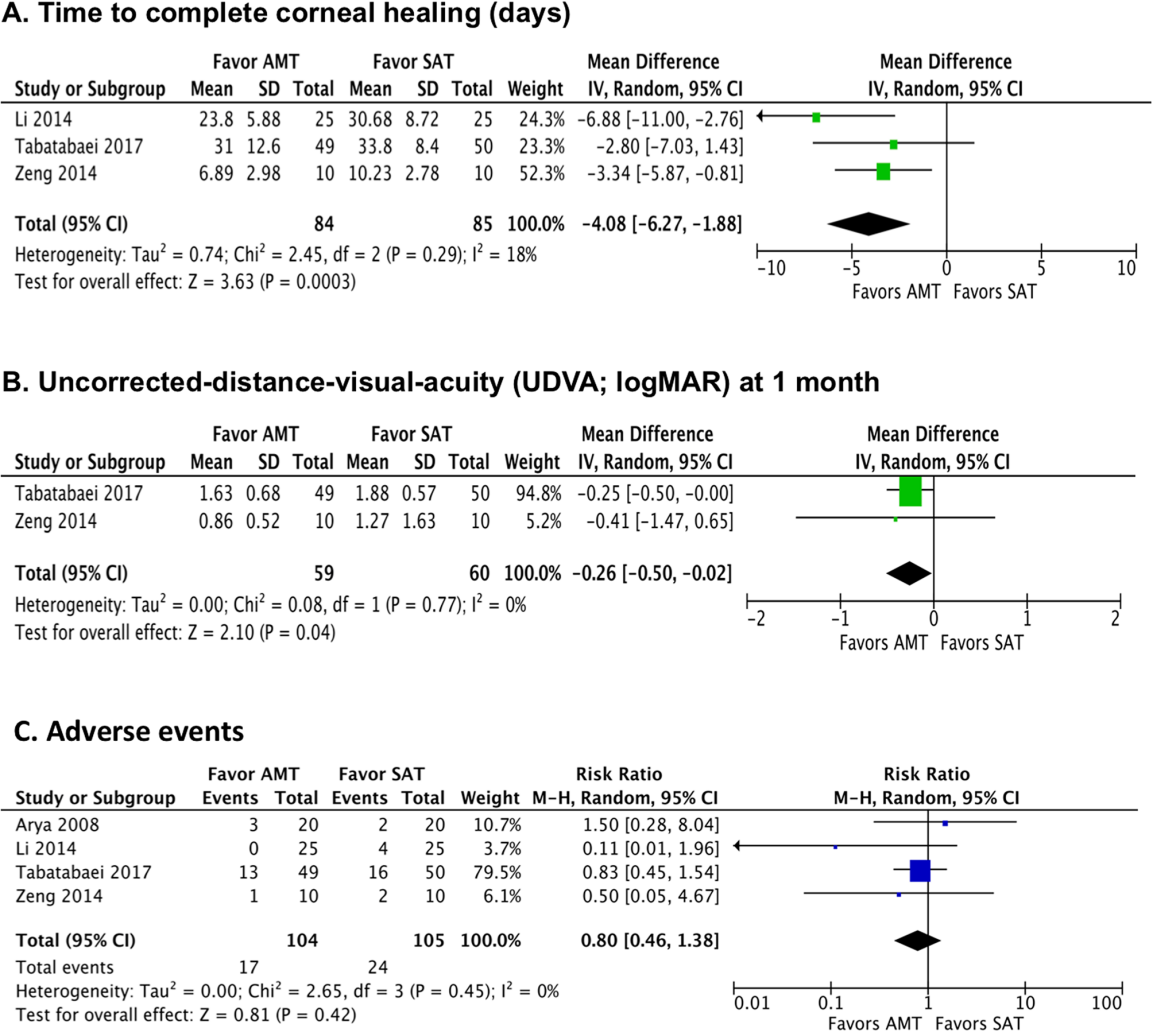
Summary of the meta-analysis (forest plot) comparing the efficacy between adjuvant amniotic membrane transplantation (AMT) plus standard antimicrobial treatment (SAT) and SAT alone in eligible randomised controlled trials, in terms of: (**A**) time to complete corneal healing; (**B**) uncorrected-distance-visual-acuity; and (**C**) risk of adverse events.

Three RCTs (n = 169 eyes) reported the primary outcome measure, which was the time to complete corneal healing^25,27,29^. There is very low-quality evidence that adjuvant AMT expedited the time to complete corneal healing compared to SAT alone [mean difference (MD) − 4.08 days; 95% CI − 6.27 to − 1.88; I^2^ = 18%; *p* < 0.001; Fig. 3A]. The quality of evidence was downgraded due to the high risk of bias and imprecision. The randomization process was not clear in one RCT^27^ and allocation concealment were not performed in two RCTs^27,29^. Furthermore, there was lack of blinding of the participants and the assessors across all three RCTs^25,27,29^. In addition, the total number of participants/eyes pooled in the meta-analysis was less than the number generated by a conventional sample size calculation.

##### Uncorrected-distance-visual-acuity (UDVA)

Two RCTs (n = 119 eyes) reported the UDVA at 1 month follow-up^25,27^. There is very low-quality evidence that adjuvant AMT resulted in better UDVA compared to SAT alone (MD − 0.26 logMAR; − 0.50 to − 0.02; I^2^ = 0; *p* = 0.04; Fig. 3B). The quality of evidence was downgraded due to high risk of bias and imprecision. The randomization process and allocation concealment were not clear in one RCT. There was lack of blinding of the participants and assessors and the sample size was limited in both RCTs. Only one RCT (n = 99 eyes) reported the UDVA at 6 months follow-up, where the adjuvant AMT group achieved a better UDVA than the SAT alone group (1.34 ± 0.69 vs. 1.69 ± 0.54; *p* < 0.001)^25^.

##### Corrected-distance-visual-acuity (CDVA)

Only one RCT (n = 99 eyes) reported the CDVA at 1–6 months follow-up, therefore meta-analysis was not possible^25^. There is low-quality evidence that adjuvant AMT resulted in better CDVA (in logMAR) at 1 month (1.44 ± 0.75 vs. 1.74 ± 0.61; *p* < 0.001), 3 months (1.23 ± 0.72 vs. 1.65 ± 0.56; *p* = 0.007), and 6 months follow-up (1.13 ± 0.68 vs. 1.55 ± 0.59; *p* < 0.001). This RCT evaluated only bacterial keratitis, but not other types of microorganism. The quality of evidence was downgraded due to some risk of bias (lack of blinding) and imprecision (limited sample size).

##### Size of ulcer or infiltrate

We did not identify any RCT that reported the size of ulcer or infiltrate. However, one RCT (n = 99 eyes) reported the area of corneal scarring (in mm^2^) at 6 months follow-up, which was found to better in the adjuvant AMT group than the SAT alone group (17.6 ± 5.7 vs. 22.8 ± 6.1; *p* < 0.001)^25^. The quality of evidence was downgraded due to some risk of bias (lack of blinding) and imprecision (limited sample size).

##### Corneal vascularization

Only one RCT (n = 99 eyes) reported the extent of corneal vascularization, measured as % of the total area of corneal surface, at 1–6 months follow-up^25^. There is low-quality evidence that adjuvant AMT resulted in less corneal vascularization (%) at 1 month (5.0 ± 4.0 vs. 7.0 ± 5.0; *p* < 0.001) and 6 months follow-up (2.0 ± 3.0 vs. 7.0 ± 6.0; *p* < 0.001). The quality of evidence was downgraded due to some risk of bias (lack of blinding) and imprecision (limited sample size).

##### Adverse events

All four RCTs (n = 209 eyes) reported the occurrence/risk of adverse events, defined as worsening of IK or perforation requiring corneal gluing or tectonic keratoplasty^25–27,29^. There is very low-quality evidence that there was no evidence of difference in the risk of adverse events between adjuvant AMT and SAT alone groups at 1–6 months follow-up (RR 0.80; 95% CI 0.46–1.38; I^2^ = 0; *p* = 0.42; Fig. 3C). The risk of adverse events was noted to be considerably variable across four RCTs, with a risk ranging between 0.0 and 26.5% in the AMT group and 10.0–32.0% in the SAT group. Such heterogeneity was likely related to the variations in the patient cohort, types and severity of IK, and the standard treatment regimen employed among different studies. For each specific complication, adjuvant AMT group had a slightly lower risk than the SAT alone group in terms of worsening of IK requiring secondary debridement (0–10% vs. 0–20%), secondary glaucoma (4% vs. 36%), and corneal perforation requiring either corneal gluing or tectonic keratoplasty (0–26.5% vs. 10–32%). The quality of evidence was downgraded due to high risk of bias and imprecision. The follow-up duration was variable across studies, ranging from 1 to 6 months. One RCT (n = 50 eyes with fungal keratitis) reported the risk of secondary glaucoma, which was 4% (n = 1 eye) in the AMT group and 36% (n = 9 eyes) in the SAT group (*p* = 0.011)^30^. According to the CONSORT reporting of harms guidance, all four RCTs were judged to be of inadequate quality as the severity of adverse events and clinical sequalae were not clearly described^25–27,29^.

### Subgroup analysis based on the type of IK

There were insufficient RCTs to perform subgroup analysis between bacterial and fungal keratitis outcomes. In view of the limitation, pooled estimates on time to complete corneal healing and adverse events were assessed based on RCTs and NCRSs. Pooled estimates of the time to complete corneal healing showed that adjuvant AMT expedited the time to complete corneal healing in bacterial keratitis (MD − 2.42 days; 95% CI − 4.53 to − 0.32; *p* = 0.02) and fungal keratitis (MD − 6.90 days; 95% CI − 11.58 to − 2.21; *p* = 0.004; Table 4). In terms of the risk of adverse events, the pooled estimates demonstrated that adjuvant AMT did not significantly influence the risk of adverse events in bacterial keratitis (RR 0.83; 95% CI 0.45–1.54), fungal keratitis (RR 0.28; 0.05–1.65), and mixed bacterial/fungal keratitis cohorts (RR 1.50; 0.28–8.04; Table 4).

**Table 4:** Pooled estimates of the time to complete corneal healing and risk of adverse events between adjuvant amniotic membrane transplantation (AMT) and standard antimicrobial treatment (SAT) alone based on comparative studies, including randomized controlled trials (RCTs) and non-randomized controlled studies (NRCS).

IK Cohort	No. of studies	Types of studies	No. of eyes	Pooled estimates (95% CI)
**Time to complete corneal healing (estimates presented in mean difference, MD)**				
Bacterial	2	1 RCT^20^	124	− 2.42 (− 4.53 to − 0.32)
		1 NRCS^23^		
Fungal	3	2 RCTs^22,33^	168	− 6.90 (− 11.58 to − 2.21)
		1 NRCS^34^		
**Risk of adverse events (estimates presented in risk ratio, RR)**				
Bacterial	2	1 RCT^20^	124	0.83 (0.45–1.54)
		1 NRCS^23^		
Fungal	2	2 RCTs^22,33^	70	0.28 (0.05–1.65)
Mixed bacteria and fungi	2	1 RCT^21^	108	1.50 (0.28–8.04)
		1 NRCS^35^		

A negative MD value indicates that adjuvant AMT group has a shorter time to complete corneal healing compared to SAT alone group.

*IK* infectious keratitis, *CI* confidence interval.

Subgroup analysis of the outcomes based on large non-comparative case series (n ≥ 5 eyes) is summarized in Table 5. Studies that reported the pooled results with no distinction made to the underlying microorganisms were excluded from the subgroup analysis. Based on these case series, complete corneal healing rate was 92.6% (113/122) in bacterial keratitis, 80.0% (96/120) in fungal keratitis, 93.8% (137/146) in herpetic keratitis, in 77.8% (7/9) Acanthamoeba keratitis, 81.8% (9/11) in mixed bacterial and fungal infection, and 100% (4/4) in mixed bacterial and herpetic infection.

**Table 5:** Summary of the healing rate and treatment failure of amniotic membrane transplantation (AMT) in large case series (N > 5 eyes) based on the types of causative microorganisms.

Authors	Year	Numbers	Complete healing	Healing time (days)	Adverse events
**Bacterial keratitis**					
Altay et al.^36^	2016	42	42 (100%)	19	0 (0%)
Berguiga et al.^37^	2013	1	1 (100%)	–	0 (0%)
Chen et al.^40^	2002	6	5 (83%)	9	1 (17%)
Eraslan Yusufoglu et al.^42^	2013	21	21 (100%)	22	0 (0%)
Gicquel et al.^44^	2007	12	12 (100%)	26	0 (0%)
Hoffmann et al.^45^	2013	3	2 (67%)	31	1 (33%)
Kim et al.^46^	2001	9	9 (100%)	–	0 (0%)
Mohan et al.^48^	2014	28	21 (75%)	–	7 (25%)
**Fungal keratitis**					
Chen et al.^39^	2006	12	11 (92%)	4–26	1 (8%)
Eleiwa et al.^41^	2020	5	5 (100%)	26	0 (0%)
Fu et al.^43^	2012	35	33 (92%)	14	1 (3%)
Kim et al.^46^	2001	2	2 (100%)	–	0 (0%)
Rao et al.^49^	2012	21	16 (76%)	–	2 (10%)
Xie et al.^54^	2014	19	8 (42%)	36	Not reported
Zhang et al.^56^	2010	26	21 (81%)	–	2 (8%)
**Herpetic keratitis**					
Altay et al.^36^	2016	42	40 (95%)	19	2 (5%)
Berguiga et al.^37^	2013	4	3 (75%)	–	1 (25%)
Eraslan Yusufoglu et al.^42^	2013	25	22 (88%)	22	3 (12%)
Hoffmann et al.^45^	2013	5	5 (100%)	21	0 (0%)
Kim et al.^46^	2001	7	7 (100%)	–	0 (0%)
Li et al.^47^	2010	18	18 (100%)	–	0 (0%)
Shi et al.^50^	2007	15	15 (100%)	15	0 (0%)
Spelsberg et al.^51^	2008	12	9 (75%)	25	3 (25%)
Wu et al.^53^	2013	18	18 (100%)	17	0 (0%)
**Acanthamoeba keratitis**					
Bourcier et al.^38^	2004	6	4 (67%)	–	0 (0%)
Kim et al.^46^	2001	3	3 (100%)	–	0 (0%)
**Mixed bacterial and fungal keratitis**					
Chen et al.^39^	2006	11	9 (82%)	7–23	2 (18%)
**Mixed bacterial and herpetic keratitis**					
Hoffmann et al.^45^	2013	4	4 (100%)	22	0 (0%)

## Discussion

To the best of our knowledge, this study represents the most up-to-date systematic review and meta-analysis examining the effectiveness and safety of adjuvant AMT for treating IK. The use of AMT for ocular diseases dates back to the early twentieth century^57^. However, the procedure was not commonly performed until 1990s where Batlle and Perdomo demonstrated the wound healing property of AM, reinvigorating the interest of utilising AMT for ocular surface disease^8^. The use of AMT for ocular surface reconstruction was subsequently popularized by Kim and Tseng^58^ using glycerin-preserved AM. Although AMT has since been performed to treat a wide array of ocular surface diseases, high-quality evidence of using AMT in treating active IK remains limited. So far, there has only been one systematic review that had partially examined the benefit of AMT in IK^24^. The systematic review included 17 studies (n = 390 eyes) and examined the healing rate and visual improvement rate after AMT in either infectious or non-infectious corneal ulcers. While the review showed that AMT was effective in treating IK, it did not answer a very important clinical question, which is whether adjuvant AMT provides any additional benefit or risk during the management of IK, in addition to standard antimicrobial treatment. In addition, the review did not capture 3 RCTs that were identified in our systematic review, rendering the evidence of their findings uncertain. Furthermore, the effect of AMT has not been examined in the context of the types of organisms.

### Summary of main findings

In this systematic review, we included a total of 28 studies (n = 861 eyes), encompassing four RCTs with 209 eyes and 24 non-RCTs with 657 eyes. Among the RCTs, the majority of the included eyes were either affected by bacterial keratitis or fungal keratitis, with only 6 eyes being affected by mixed bacterial and fungal keratitis. None of the RCTs examined the effect of adjuvant AMT in herpetic or Acanthamoeba keratitis. Based on the meta-analysis of three RCTs, we demonstrated that early adjuvant AMT shortened the time to complete corneal healing by approximately 4 days when compared to SAT alone. In addition, it was shown that IK patients treated with adjuvant AMT achieved 0.26 logMAR (equivalent to 2–3 Snellen lines) better UDVA at 1 month follow-up compared to SAT alone. No evidence of difference was noted in terms of the risk of adverse events. Furthermore, other beneficial effects such as better CDVA and less corneal vascularization were shown in the adjuvant AMT group.

The observed beneficial effects in the adjuvant AMT group are likely attributed to the dual antimicrobial and anti-inflammatory properties of AMT^8,16^. The plausible mechanisms of antimicrobial effect of AMT in IK are at least twofold. First, the antimicrobial activity is directly linked to the presence of various antimicrobial components, including lysozyme, transferrin, and immunoglobulin, in the amniotic fluid^8,59^. Second, studies have shown that AM could serve as an effective antibiotic reservoir when used in combination with antibiotics and provide sustained drug delivery^60^. Furthermore, AM has been shown to exhibit anti-inflammatory function via regulation of T-cell function and secretion of anti-inflammatory antagonists, including IL-1ra, sTNF, and VEGF-R^61,62^. This anti-inflammatory property will have a long-term beneficial effect on the cornea, hence vision, as persistent corneal vascularization could negatively affect the vision, either directly via encroachment on the visual axis or indirectly via lipid keratopathy where exudation of lipid and inflammatory cells extends to the visual axis^63^. Moreover, it increases the risk of graft rejection should corneal transplantation needs to be carried out to restore the optical clarity of the cornea^63,64^.

Although the benefit of AMT in herpetic keratitis has not been ascertained in RCTs, our systematic review (based on large case series) showed that it enabled a high rate (94%) of complete corneal healing. Herpetic keratitis, particularly the necrotizing form, is a potentially sight-threatening condition that is difficult to treat clinically^65^. It is also notoriously known to be associated with neurotrophic keratopathy, which results in delay in corneal healing^65,66^. Therefore, AMT serves as a useful adjunct treatment in this clinical circumstance in view of its dual anti-viral and wound healing properties^67,68^. On the other hand, only two case series have reported the use of AMT in Acanthamoeba keratitis, albeit good effect was observed. The broad-spectrum antimicrobial activity of AMT (observed in our systematic review) against a wide range of microorganisms is particularly beneficial in the management of IK as polymicrobial keratitis is a relatively common entity, which often poses significant diagnostic and therapeutic challenges^4,5,69^. Based on large case series, adjuvant AMT has been shown in our review to be an effective treatment for mixed bacteria/fungal keratitis (82% complete healing; n = 9/11) and mixed bacterial/herpetic keratitis (100% complete healing; n = 4/4).

A number of studies have also demonstrated that AMT, when employed as multi-layer, could effectively treat IK with threatened or actual corneal perforation (up to 5 mm)^35,37,43,52^. Studies have demonstrated that multi-layer AMs can promote re-epithelialization of the cornea and integrate with the corneal stroma, with progressive repopulation of AM by cornea stroma-derived cells, ultimately resulting in corneal healing and thickening^70^. This approach helps obviate the need for an emergency therapeutic keratoplasty, which is known to be associated with high risk of graft failure (in the setting of active inflammation) and recurrence of infection^69,71,72^. It would also help reduce the burden on the availability of donor corneas, which have been significantly affected by the recent COVID-19 pandemic^73^.

### Overall completeness and applicability of evidence

This study represents the most up-to-date systematic review and meta-analysis specifically examined the effectiveness and safety of adjuvant AMT in managing all types of IK. The four RCTs included primarily bacterial and fungal keratitis cases, therefore the outcome of the meta-analysis should be interpreted in the context of these types of infection. All RCTs were completed without any significant drop out and there was no concern with the safety of AMT.

The reporting and categorization of the baseline severity of IK was considerably variable among the RCTs. Two studies included corneal ulcers with a mean diameter of 5–7 mm (severe disease)^25,27^, one study included mainly moderate (2–5 mm in diameter) and severe (> 5 mm in diameter) ulcers, and one did not report the baseline severity. Such heterogeneity highlights the need for standardized reporting and a core outcome set in the future clinical trials related to IK. Of the three RCTs that reported the baseline severity of IK, the majority of the included participants/eyes were affected by moderate-to-severe IK, suggesting that adjuvant AMT would be useful for this group of patients. Future studies examining the effectiveness and safety of adjuvant AMT in mild-to-moderate IK would be required before being routinely applied in clinical practice.

Another aspect that was not examined in this systematic review is the effects of different surgical techniques and types of AMT on the clinical outcomes. In clinical practice, a number of AMT techniques have been described. It can be used as a patch (or overlay) where an AM that is transplanted to cover the ocular surface, allowing the host corneal epithelium to regenerate under the AM. It can also be used as graft (or inlay) where a smaller AM is grafted over the corneal epithelial defect and act as a substrate for the host epithelium to grow over. Occasionally, a sandwich AMT technique, utilizing a mixture of overlay and inlay, is performed to facilitate the regeneration of host epithelium between the AMs^16^. In addition, various forms of AMs, either fresh form, cryopreserved, lyophilized (freeze-dried), or air-dried AM, have been used, with comparable clinical efficacy observed amongst them^16^. While studies have shown the varying effects of preservation method on the structural and functional properties of AMs, strong clinical evidence is lacking in the literature. Cryopreserved AMs, depending on the duration of the preservation, have been shown to possess no or less viable cells compared to fresh, freeze-dried or vacuum-dried AM, which have more viable cells with proliferative capability^16,74^. On the other hand, some studies have demonstrated that lyophilization of the AMs may lead to a greater reduction in the growth factors compared to cryopreservation. Preclinical studies observed similar efficacy in terms of healing activity between cryopreserved and freeze-dried AMs, supporting the use of both types of AMs in clinical setting^75,76^. In our systematic review, the three RCTs included in the meta-analysis for time to complete healing outcome, one RCT employed cryopreserved AMT, another RCT utilized freeze-dried AMT and the remainder did not report the preservation method used. Therefore, it is not possible to perform a head-to-head comparison between each type of AM due to limited studies, but the overall effect of either type was positive.

### Quality of evidence and potential biases in the review

Similar outcome reporting in the included RCTs has enabled the meta-analysis, which demonstrated the value of adjuvant AMT in expediting complete corneal healing, reducing corneal vascularization and potentially achieving better visual outcome. However, two RCTs have some concerns on the risk of bias and another two RCTs have high risk of bias. Due to the lack of information in the randomization process, lack of blinding, and under-powered sample size. In addition, none of the RCTs were prospectively registered with any clinical trial database, which limits the assessment of selective reporting of the outcomes.

Of all four RCTs, only Tabatabaei et al.^25^ conducted a sample size calculation based on the anticipated difference in the size of corneal scar (mean effect size of 4 mm^2^, standard deviation of 6 mm^2^, and power of 90%), which yielded a sample size of 48 eyes in each group. However, it is noteworthy to mention that size of corneal scar is not a routine outcome measure for IK. Based on the findings of our systematic review, an appropriate sample size (using time to complete corneal healing as the main outcome measure) is estimated to be at approximately 200 participants/eyes (based on a mean difference or effect size of 4.08 days, standard deviation of 14.56 days, power of 80% and alpha of 5%). This sample size is also supported by some previous RCTs examining the effectiveness of therapeutic CXL (or PACK-CXL) for IK, where an appropriate sample size was estimated at around 200–250 participants, using time to complete corneal healing as the main outcome measure^10,77^. This large sample size would then potentially control for various potential confounding variables that could affect the time to complete healing, including the demographic factors, initial size and severity of the corneal ulcer, types of organisms, and underlying comorbidity (e.g. diabetes).

Another important bias, which is a common issue in surgical trial, is related to the difficulty in blinding (or masking) the participants and the surgeons^78,79^. This is evident in our systematic review where none of the RCTs had implemented or reported any measure to ensure the masking of participants or surgeons, which could potentially lead to risk of bias. In a trial, masking can be introduced to 5 groups of individuals, including participants, surgeons, data collector, outcome adjudicators and data analysts. While it is always possible to mask the latter three groups (i.e. data collectors, outcome adjudicators and data analysts), masking of the participants or surgeons may not always be possible, depending on the surgical intervention and design. For instance, in AMT trial, it would have been difficult to mask the patients or surgeons as the patients would have known that an AMT has been performed (unless a sham surgery is performed) and the surgeons would have seen the physical presence of the AM postoperatively. However, masking the other 3 groups of individuals could reduce the bias. As fewer than 10 studies were eligible for inclusion, we were unable to use a funnel plot to identify possible publication bias.

In conclusion, this systematic review and meta-analysis demonstrated the benefit of early adjuvant AMT in accelerating corneal healing and improving visual outcome in moderate-to-severe bacterial and fungal keratitis. However, further adequately powered and well-designed RCTs are required to ascertain the true potential of adjuvant AMT in treating active IK, particularly herpetic and Acanthamoeba keratitis. Future standardization of the baseline assessment and core outcome set for clinical trials related to IK would also be invaluable.

## Methods

### Protocol registration

The systematic review protocol was registered with PROSPERO (registration number: CRD42020175593) and The Joanna Briggs Institute of Evidence Synthesis^80^.

### Data sources and search methods

Two authors (D.S.J.T and C.H.) searched MEDLINE (January 1950 to November 2020), EMBASE (January 1980 to November 2020), Cochrane Central Register of Controlled Trials (CENTRAL), ISRCTN registry (www.isrctn.com/editAdvancedSearch), US National Institutes of Health Ongoing Trials Register ClinicalTrials.gov (http://clinicaltrials.gov) and World Health Organization (WHO) International Clinical Trials Registry Platform (ICTRP) (www.who.int/ictrp) for primary research related to AMT for IK. There was no date or language restriction in the search for trials. Electronic databases were first searched on 01 March 2020, followed by a final update on 01 November 2020. The bibliographies of the included articles were reviewed for any additional eligible articles. Key words used were “amnion”, “amniotic membrane”, “corneal ulcer”, “corneal infection”, “infectious keratitis”, and “microbial keratitis”. Search strategies for MEDLINE and EMBASE are provided in the Supplemental Table S1.

### Study selection

All clinical studies, encompassing RCTs, NRCSs and case series (n ≥ 5 eyes), related to AMT for IK were included as few RCTs were anticipated. The analysis was conducted at two levels; (1) a meta-analysis of all eligible RCTs and (2) a systematic review of all clinical studies, including NRCSs and case series. All types of IK, including bacterial, fungal, viral, parasitic or mixed infection, were included in this review. Studies that evaluated cases of non-infectious keratitis, other types of surgical interventions, or AMT used for non-antimicrobial purpose were excluded from this study. Small case series (N < 5 eyes), reviews, published abstracts, laboratory and animal studies were also excluded. For the meta-analysis, the intervention group included cases of IK that were treated by AMT and SAT whereas the control group included cases of IK that were treated with SAT alone. There was no restriction applied to the published language, location or setting of the study, or patient demographic factors. This study conformed to the Preferred Reporting Items for Systematic reviews and Meta-Analysis (PRISMA) guideline (see Supplementary Table S2)^81^.

### Data extraction

A web application designed for systematic reviews, Rayyan (Qatar)^82^, was used to help collate the potential studies and expedite the initial screening of abstracts and titles. The titles and abstracts obtained from the searches were independently screened by two authors (D.S.J.T. and C.H.) to include studies that fulfilled the eligibility criteria. The authors then independently assessed the full-text version of all the selected articles and extracted data onto a standardized data collection form for qualitative review. The extracted data included the authors, year of publication, sample size, types of interventions, types of causative microorganisms, outcomes and complications^80^. Discrepancies were resolved by group consensus if consensus could not be reached.

For the meta-analysis, the following information were extracted from the included RCTs and entered into RevMan (Review Manager 5.4) software^80,83^:

1. Study characteristics: Year of publication, country of study, prospective registration of clinical trials in a publicly accessible database, sample size, eligibility criteria, demographic factors, diagnostic criteria, method of randomization, method of masking, number of study arms, number of participants, types and techniques of AMT, types of comparators, source of funding, and any potential conflict of interest. Types of AMT were divided based on the preservation technique, which included fresh, cryopreserved, freeze-dried (or lyophilization), air-dried and others. Surgical techniques were categorized based on the number of AMT (i.e. single vs. double vs. multiple layers) and the type of transplant (i.e. overlay/patch vs. inlay/graft vs. mixed overlay and inlay).
2. Outcomes: Primary and secondary outcomes (including the time point at which the outcomes were assessed), intra- and post-operative complications, adverse events, need for secondary surgery, duration of follow-up, and rate of loss to follow-up.

### Outcome measures

For the meta-analysis, the primary outcome measure was the time to complete corneal healing (defined as complete corneal re-epithelialization and clearance of infiltrate and hypopyon; days) and the secondary outcome measures included the corrected-distance-visual-acuity (CDVA; logMAR) and uncorrected-distance-visual-acuity (UDVA; logMAR) at final follow-up (1–6 months), size of corneal ulcer (at 1–6 months), extent of corneal vascularization, and risk of adverse events (defined as worsening IK and/or corneal melt/perforation requiring gluing or tectonic/therapeutic keratoplasty or evisceration) at final follow-up (1–6 months). These outcome measures were similarly used in our previous systematic review and meta-analysis in examining the effectiveness and safety of adjuvant PACK-CXL for IK^10^. Relevant data of all included studies were summarized and reported.

Continuous variables such as time to complete corneal healing, UDVA, CDVA, and size of corneal epithelial defect and infiltrate, were presented as mean with standard deviation (SD). In studies that reported median and interquartile range, the means and SDs were estimated using formulas reported by Wan et al.^84^ and the Cochrane Handbook estimator^85^. Dichotomous variable such as risk of adverse events was defined by the number of eyes with adverse events.

### Assessment of risk of bias

Risk of bias was assessed by two authors (D.S.J.T. and C.H.) independently and any disagreement was adjudicated by group consensus. Included RCTs were assessed for sources of systematic bias using the revised Cochrane risk of bias tool (RoB 2 tool)^86^. The review authors were not masked to the authors of the studies during this assessment. A judgement of risk of bias as ‘high risk’, ‘some concern’, or ‘low risk’ was made for the following domains: (1) randomization process; (2) deviations from intended interventions; (3) missing outcome data; (4) measurement of the outcome; and (5) selection of the reported result^86^. NRCSs were assessed for risk of bias using the ROBINS-I tool^87^ against seven domains; the worst judgement in any of the domains was used as the overall risk of bias.

### Measure of treatment effect

Dichotomous data were measured as risk ratios (RRs) with 95% confidence intervals (CI) and continuous data as mean differences (MDs) with 95% CI^80,88^. The unit of analysis was the eye as the patient might have different causes of infection and treatment in each eye. There was no issue with the unit of analysis in the included RCTs. The review was conducted based on the available data from the trials. When data were unavailable but the level of missing data and reasons for missing data in each group were similar, data were analyzed even when intention-to-treat (ITT) analysis was not performed.

### Assessment of heterogeneity

The heterogeneity of the RCTs and NRCSs was checked by careful review of the full-text, assessment of forest plots and examination of the I^2^ value with its confidence interval. The overall characteristics of the studies, in particular the types of participants, causes of IK and types of interventions were examined to assess the extent to which the studies were similar enough to make pooling study results sensible^80^. The results of forest plots were reviewed for consistency of the size and direction of effects. I^2^ values greater than 50% were considered indicative of substantial heterogeneity and meta-analysis could not be conducted due to inconsistency of effect estimates^85^. Random-effects model was used for the meta-analysis as some degree of heterogeneity will always exist due to clinical and methodological differences of the studies^80^. The Chi^2^ *p* value was also considered as this has a low power when the number of studies were few. A *p* value of < 0.1 was considered statistically significant^85^.

### Data synthesis and analysis

A meta‐analysis was undertaken when there were sufficient similarities in the reporting of outcome measures^80^. A random‐effects model in RevMan 5.4 was used in view of the expected heterogeneity across different studies. The Mantel–Haenszel method was employed for analysing the risk ratio (RR) of adverse events in view of the small expected number of events^80^. If there was inconsistency between the results of individual studies such that a pooled result might not be a good summary of the individual trial results—for example, the effects were in different directions or I^2^ > 50% and *p* < 0.1—the data were not pooled but described in narrative format^80,88^. Where there was statistical heterogeneity the data were pooled when all the effect estimates were in the same direction, such that a pooled estimate would seem to provide a good summary of the individual trial results. Sensitivity analysis was performed by assessing the impact of including studies at high risk of bias for an outcome in one or more key domains^80^. This was conducted by omitting each study in turn to examine the influence of individual studies (with high risk of bias) on the overall pooled estimate. A summary of findings is presented below including the assessment of the quality of the evidence for outcomes using the GRADE approach with GRADE Pro/GDT software^89^. All RCTs were started with a rating of ‘high-quality’ evidence and were downgraded by one level for serious concerns (or by two levels for very serious concerns) regarding the risk of bias, inconsistency, indirectness, imprecision and publication bias. The quality of evidence of studies was graded by two assessors (D.S.J.T and C.H.) independently and any disagreement was adjudicated by group consensus.

### Subgroup analysis based on the type of organisms

In addition to the meta-analysis, a subgroup analysis of the effectiveness and adverse events based on different types of organisms was performed. In view of the anticipated low number of RCTs, both experimental and quasi-experimental study designs including RCTs and NRCSs were included in the subgroup analysis. Descriptive case series were also reviewed for rare or uncommon adverse events. Pooled estimates of the time to complete corneal healing and risk of adverse events across comparative studies, including RCTs and NRCSs, were calculated. Causes of IK were categorized as bacterial, fungal, viral, Acanthamoeba, or mixed infection.

## Supplementary Information

Supplementary Table S1.

Supplementary Table S2.
