# Supplementary material for: Amniotic membrane transplantation for infectious keratitis: a systematic review and meta-analysis
Source: Sci Rep. 2021 Jun 21;11:13007. doi: 10.1038/s41598-021-92366-x (PMC8217254; doi:10.1038/s41598-021-92366-x)
Supplement: Supplementary file 1 — Supplementary Table S1. [file 41598_2021_92366_MOESM1_ESM.docx]

**Supplementary Table S1.** Search strategy for amniotic membrane transplantation for infectious keratitis.

| **Search strategy for MEDLINE**   1. Keratitis.mp 2. Corneal infect*.mp 3. Corneal ulcer*.mp 4. Exp Keratitis/ 5. Exp Corneal Ulcer/ 6. 1 or 2 or 3 or 4 or 5 7. Exp Amnion/ 8. Amnion.mp. 9. Amniotic membrane.mp. 10. 7 or 8 or 9 11. 6 and 10 12. Limit 11 to humans |
| --- |
| **Search strategy for EMBASE**   1. Keratitis.mp 2. Corneal infection.mp 3. Corneal infections.mp 4. Corneal ulcer*.mp 5. Exp keratitis/ 6. Exp bacterial eye infection 7. Exp amnion/ 8. Amniotic membrane.mp 9. 1 or 2 or 3 or 4 or 5 or 6 10. 7 or 8 11. 9 and 10 12. Limit 11 to humans |
